# Cutaneous Manifestations of COVID-19 in the Lower Limbs: A Narrative Review

**DOI:** 10.3390/ijerph19148861

**Published:** 2022-07-21

**Authors:** Inmaculada C. Palomo-Toucedo, Manuel Jesús López-Sánchez, María Reina-Bueno, Manuel Coheña-Jiménez

**Affiliations:** 1 Department of Podiatry, Faculty of Nursing, Physiotherapy and Podiatry, University of Seville, 41009 Seville, Spain; ipalomo@us.es (I.C.P.-T.); mcohena@us.es (M.C.-J.); 2 Independent Researcher, 41009 Seville, Spain; manuejls@outlook.es

**Keywords:** COVID-19, SARS-CoV-2, skin manifestations, chilblain, acro-ischemic lesions, cutaneous manifestations

## Abstract

In 2020, the World Health Organization declared the COVID-19 pandemic. This infectious pathology can be associated with different manifestations in different body systems, among which are dermatological lesions. The purpose of this work is to determine the most frequent dermatological signs, in the lower limbs, produced by SARS-CoV-2. To carry this out, a bibliographic search was performed in the following databases: PubMed, SciELO, ScienceDirect, Cochrane Database of Systematic Reviews, and the Google Scholar literature. The inclusion criteria were articles that included confirmed subjects or those with a clinical suspicion of COVID-19, written in the Spanish or English languages, and the results presented clinical manifestations in the lower extremities. Initially, 128 scientific documents were identified and, after reading the title and abstract, 18 articles were selected. The most frequent skin lesions on the lower limbs are acral lesions such as pernio erythema or ischemic lesions, maculopapular rash, petechiae, and erythematous plaques.

## 1. Introduction

In 2020, the World Health Organization (WHO) declared the Coronavirus Disease-19 (COVID-19) pandemic, caused by infections with a new pathogen called Severe Acute Respiratory Syndrome CoronaVirus-2 (SARS-CoV-2) [1]. This disease has become a global health problem that is declared a worldwide Public Health Emergency, which, as in previous coronavirus infections, has led to Severe Acute Respiratory Syndrome (SARS). There are numerous identified coronaviruses, some of which cause the common cold and some of which the general population is immunized against [2]. These infections have a high mortality rate, as was the case in China in 2002, with a 10% fatality rate, or in Saudi Arabia in 2012 with a 35% mortality rate, although the WHO estimates that these figures could double or triple if unreported cases are taken into account. This infection has almost spread to the entire world [3]. In Spain, the case fatality rate has been very different depending on the geographical area [4]. The severity of the disease will depend on several factors: intrinsic factors, such as susceptibility and virulence, and extrinsic factors such as healthcare, demographics, and treatment. These loosely defined and homogenous severity criteria varied as the pandemic and knowledge of the disease have progressed [5].

**Figure 1:**
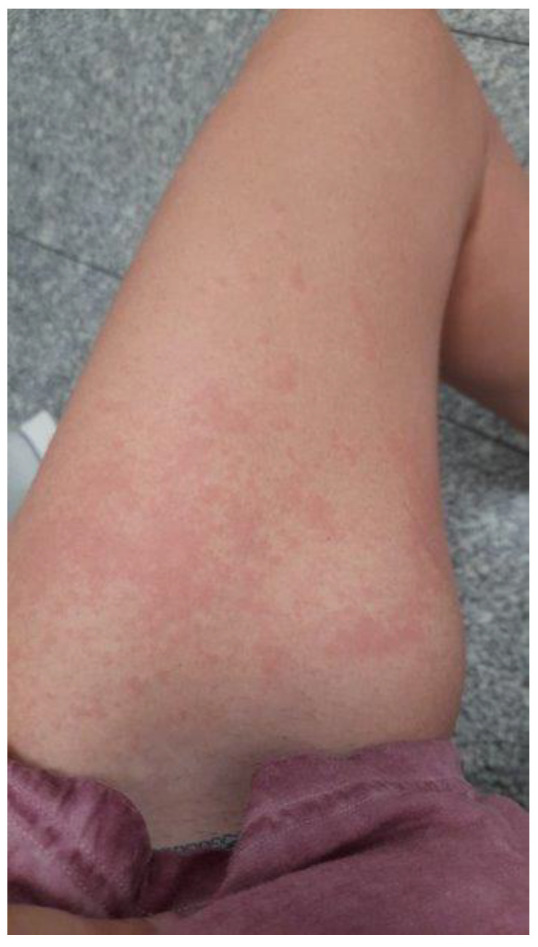
Woman aged 33-year-old with COVID-19 and skin lesion (exanthema, urticarial, and maculopapular lesions) on left lower limb.

Infections with SARS-CoV-2 have general manifestations ranging from very mild symptoms such as fever, dry cough, dyspnea, gastrointestinal symptoms, anosmia, and odynophagia to severe complications such as bilateral pneumonia and respiratory failure [6]. The skin is one of the organs most affected by COVID-19 [7]. Skin lesions are described as milder signs of the disease, although they were often the only manifestations of infection. Skin lesions, as seen in Figure 1, can lead to the diagnosis of COVID-19 in asymptomatic patients [8].

Previous studies have described the clinical and dermatological manifestations in the feet, the most frequent being oedema, exanthema, erythema pernio (also called chilblains), ischaemia, distal necrosis, vesicles, maculopapules, papulosquamous lesions, urticaria, and recurrent herpes [9].

**Figure 2:**
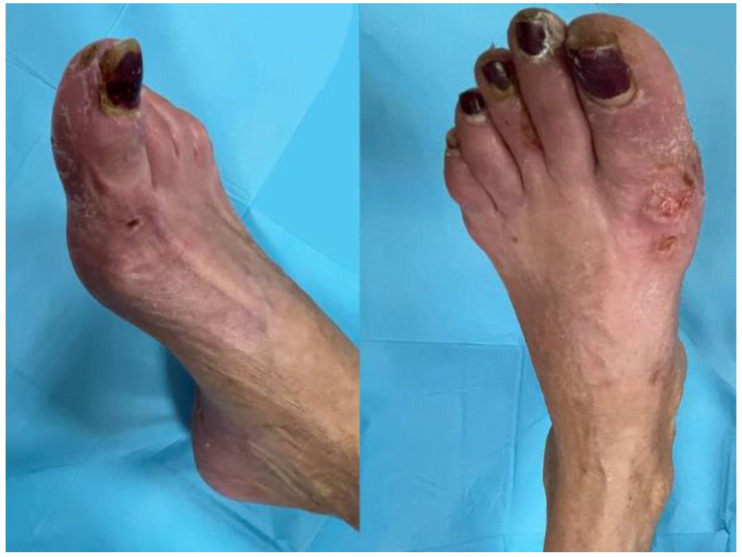
Woman aged 54-year-old with acral lesions caused by COVID-19 on both feet (blisters on the toes similar to perniosis).

Patchy acrocyanotic lesions have been sometimes described with blisters on the fingers and toes, similarly to perniosis and occurring in children and adolescents without other symptoms [10]. These apparently less-severe dermatological signs may be directly associated with the disease and are, therefore, of the utmost importance for healthcare professionals to detect, given that the incidence of dermatological signs of COVID-19 in December 2020 was between 0.2% and 29%, even in undiagnosed patients [8]; see Figure 2.

**Figure 3:**
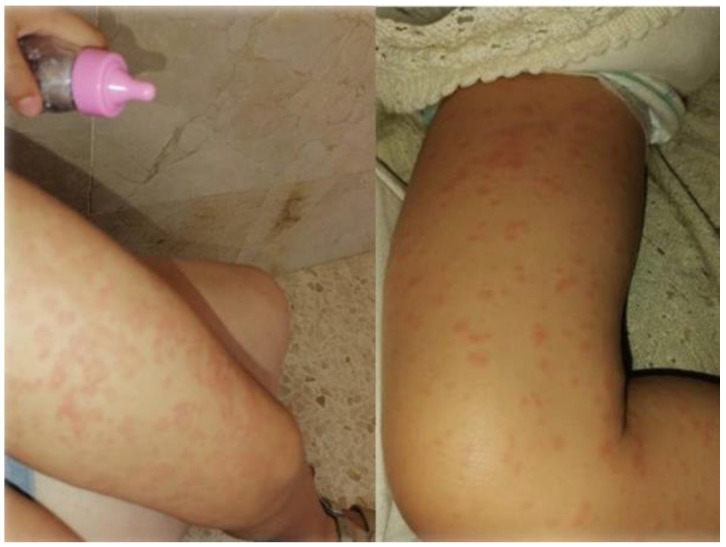
Girl aged 6-year-old with COVID-19 and with lower limb erythema (maculopapular rash with pruritus).

In SARS-CoV-2, as in other viral diseases, a maculopapular rash may appear with or without pruritus, which is the most frequent dermatological manifestation caused by COVID-19. The rashes are mostly present in women and are located on the trunk. It has been reported that, although asymptomatic in 35% of patients, pruritus was the most frequent symptom, occurring in 57% of cases. This type of rash is mainly seen in the active phase of the disease [11]. Treatment is usually symptomatic and will vary depending on the type of maculopapular rash. It is also important to make a differential diagnosis with drug reactions or other types of viruses [12]; see Figure 3.

The second most frequent rash in patients with COVID-19 is urticaria, which is more frequent in women (66%) of an average age (47.6 years). Urticaria is the presence of raised, reddish wheals that are often pruritic and appear in different circumstances, and may even be idiopathic [13]. The highest number of cases was reported in Spain, occurring mainly during the active phase of the infection [11]. Erythema pernio is the oedematisation of the smaller blood vessels that innervate the skin, resulting from vasoconstriction and hypoxaemia caused by prolonged exposure to cold and then to sudden heat. Footcare professionals should establish a differential diagnosis with Raynaud’s syndrome, acrocyanosis, vasculitis, lupus erythematosus, *livedo reticularis*, and significant ascorbic acid deficiencies [14]. There is no consensus among authors regarding the manifestation of *livedo reticularis*, which is a rash characterised by a bluish-red mottling of the skin in the form of a web. There is an idiopathic form that particularly affects the legs, thighs, and buttocks, which is accentuated by cold and is more common among women under 40 years of age [14].

Recently, Galván et al. 2020 reported five main clinical patterns associated with COVID-19: acral lesions of erythema with vesicles or pustules (pseudoperniosis) (19%), vesicular lesions (9%), urticarial (19%), maculopapular (47%) and livedo, and necrosis (6%) [15]. Some of them have been described and classified within the prevalence of dermatological manifestations associated with COVID-19 by other authors [16].

The aim of this work has been to analyse and synthesise the current state of knowledge on the localised dermatological manifestations on the feet of patients with COVID-19 using a narrative review.

## 2. Materials and Methods

A review of the existing scientific literature on the dermatological clinical manifestations of COVID-19 in the feet was conducted. The main electronic databases were analysed: PubMed, SciELO, ScienceDirect, Cochrane Database of Systematic Reviews, and Google Scholar grey literature. Keyword searches were assigned to Medical Subject Headings (MeSH). All the included studies were published between 2020 and 2021. The following search strings were used: (COVID-19 AND lower limbs); ((SARS-CoV-2) AND dermatology); ((SARS-CoV) AND epidemiology); (COVID-19 AND lower limbs AND dermatology); (COVID-19 AND epidemiology); (COVID-19 AND injury); (COVID-19 AND foot); (COVID-19 AND foot AND dermatology); (SARS-CoV-2) AND foot); (skin AND COVID-19). The searches were conducted in October 2021, after building the strategies used in the different databases.

Inclusion criteria. A review methodological design was chosen to summarise and analyse the available evidence on this topic. A narrative review and case studies, including observational studies, were conducted. We considered those in which participants were symptomatic or asymptomatic subjects confirmed with COVID-19 or with clinical suspicion of COVID-19. Reviews were required to be published in English or Spanish. Outcomes presenting clinical manifestations in the lower limbs of patients with COVID-19 were considered. All lower limb signs and symptoms were eligible for this review.

Exclusion criteria: Studies that did not meet the pre-defined criteria mentioned above and were not conducted in humans.

Assessment of the characteristics of review studies. Two independent reviewers carried out the selection procedure. Each reviewer read the title and abstract of each paper and assessed whether each study met the inclusion criteria. The papers chosen by each of the reviewers were again screened separately. In the full reading, they were once more assessed for their suitability according to precise compliance with the defined inclusion criteria, which was necessary for the final choice of study for the review. In the data extraction and to simplify the selection procedure, due to the high number of references analysed, an Excel page was designed as a data collection form in which inclusion was recorded by coding criteria. Inclusion or exclusion was discussed by the two initial reviewers, but sometimes, the mediation of a third reviewer was required to decide the final inclusion or exclusion of some of the papers due to the absence of agreement between the first two reviewers.

## 3. Results

**Figure 4:**
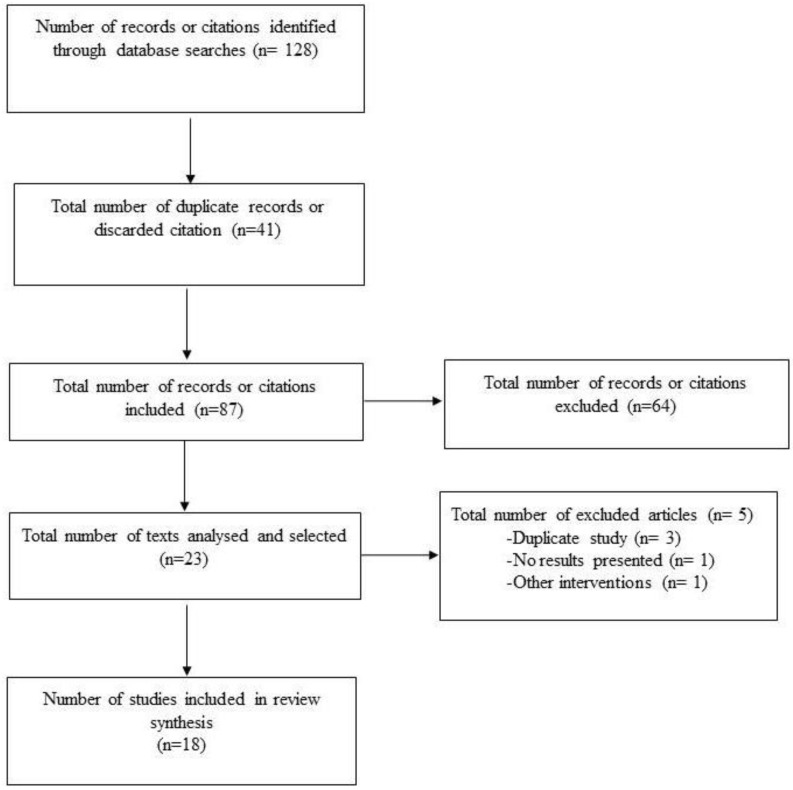
Different stages in the selection process of the studies involved.

Figure 4 shows the flow chart used to represent the different phases of the selection procedure of the studies finally included in the review.

Of the 18 included studies, 4 were cross-sectional studies, 6 others were case series studies, 2 were case reports, and the other 6 were narrative reviews. The total number of participants in all the studies was 1925 (1024 men and 901 women; six studies did not specify the sex of the participants). Ages ranged from 2 to 70 years. The studies were conducted on children, adolescents, and adults. To better present the analysis of the studies, these were categorised according to the clinical manifestations in the feet. Table 1 presents the main characteristics of the studies and the most significant data extracted in this review. This section may be divided into subheadings. It should provide a concise and precise description of the experimental results, their interpretation, as well as the experimental conclusions that can be drawn.

## 4. Discussion

The rapid evolution of the pandemic caused by SARS-CoV-2 infection and its first appearance meant that the skin findings which patients presented with went unnoticed [17]. Depending on the type of dermatological manifestation, this can be associated with a type of disease severity, which is of great prognostic value. Thus, by the end of 2020, the incidence of dermatological lesions caused by COVID-19 ranged from 0.2% to 29% and was important in the diagnosis of asymptomatic patients. These skin lesions included maculopapular, urticarial, vesicular, and chilblain-like lesions [8]. Other forms of skin lesions were associated with vasculopathy, vasculitis, and thrombotic vasculopathy [7].

We now know that the disease does not manifest itself with the same severity in all patients and that, in the mildest cases, one of the most frequent manifestations was the presence of blistering lesions or chilblains on the feet. This fact calls for further studies by foot health professionals to assist in the diagnosis and early detection in some cases of mild COVID-19 disease.

According to the published literature, there is no clear consensus on the nomenclature of certain skin manifestations, which may hinder the prognosis of COVID-19 disease; it is, therefore, essential to standardise the nomenclature and classify skin lesions in a uniform manner [15,16,28]. The great disparity in the data provided by each study did result in difficulties when analysing the literature and writing this review. Similarly, most studies do not differentiate the results by sex, with the exception of Marzano et al., 2020, who only studied a sample of men [24].

Regarding the prevalence, frequency of occurrence, and type of lesions associated with COVID-19, there are differences according to the authors consulted. Some of the most frequent early skin lesions detected in Italy were erythematous eruptions [18]. Estébanez et al. (2020) described confluent erythematous, yellowish papules as the first to appear and the most frequent [19].

Specifically, on the feet, the presence of chilblains has been detected much more frequently in consultations during the post-pandemic period. Cases of perniosis diagnosis have increased since 2020 and are directly linked to COVID-19 [18,22]. What several authors agree on is that the dermatological signs possibly associated with SARS-CoV-2 infection are cases of acral lesions, specifically perniosis or “COVID toes”. The most frequent location was on the lower limb and in acral areas such as the feet [15,21,23,27,28,29]. Disagreeing with these authors, Carrascosa et al. report a higher frequency of maculopapular rashes located on the trunk [16]. There is some disparity with regard to the age of onset; chilblains have been reported both in young ages [23] and in older ages associated with severe COVID-19 disease [18].

The pharmacological treatments used for dermatological conditions linked to COVID-19 ranged from initial analgesic and antihistamine treatments to the use of local glucocorticoids, and even misdiagnosis and treatment with oral macrolides and topical therapy [19,20,30]. In very severe cases involving dry gangrene and acrocyanosis, treatment with anticoagulants has been effective, although mortality remains high [25].

## 5. Conclusions

The most common skin lesions on the lower limbs are acral lesions such as erythema pernio, maculopapular rash, petechiae, and erythematous plaques. Ischemic phenomena are indicative of more severe disease diagnosed in a hospital setting. Age, sex, viral load, etc., influence the systemic and local manifestations and, thus, the skin lesions diagnosed. The scientific community is still investigating methods for improving treatments and unifying criteria, which, except for infection prevention, do not exist. There are some foot-related skin manifestations that health professionals should be aware of for early detection. There is not yet clear evidence of a direct association between SARS-CoV-2 and skin lesions on the feet.

## Figures and Tables

**Table 1:** Main characteristics of the included studies.

	Type of Study Participants (n)	Average Age (Years) Men Women	Aim	Types of Skin Manifestations on the Foot
Guan 2020 [17]	Cross-sectional study (n = 1099)	47 M = 637 W = 462	To describe the clinical characteristics of patients with COVID-19	Not specified. Skin findings were missed on initial COVID-19.
Recalcati 2020 [18]	Case series study (n = 107)	72 M = 58 W = 49	To summarise perniosis-like dermatological lesions in young patients with COVID-19.	Acrocyanosis due to respiratory failure. Foot thrombosis. Maculo-papular lesions on feet and hands. 90% of the lesions were located on the fingers and toes and 81.8% were located on the feet.
Estébanez 2020 [19]	Report case (n = 1)	28 M = 0 W = 1	To describe a new skin manifestation on the heel of the foot caused by COVID-19.	Dermatological sign of confluent erythematous-yellowish papules on the heels. (COVID-19 positive).
Carrascosa 2020 [16]	-	-	To summarise the prevalence of dermatological manifestations associated with COVID-19.	Acral lesions, vesicular rashes, urticarial rashes, maculopapular rashes, and livedoid/necrotic lesions.
Lu 2020 [13]	Case series study (n = 3)	M = 2 W = 1	To report clinical features on the foot in a quasi-symptomatic patient.	Urticaria on the lower extremities, with no other symptoms associated with COVID-19.
Henry 2020 [20]	Report case (n = 1)	27 M = 0 W = 1	To report a case of onset of urticarial rash before the onset of fever or respiratory symptom after diagnosis of COVID-19.	Pruritic rash of disseminated erythematous plaques with facial and acral involvement.
Masson 2020 [21]	Retrospective cross-sectional study (n = 277)	27 M = 114 W = 113	To analyse the dermatological manifestations associated with COVID-19.	Urticaria (n = 26); vesicular lesion (n = 41); acral lesion (n = 142); morbilliform lesion (n = 25); petechiae (n = 7); livedo. Reticularis (n= 4); and other types of dermatological lesions (n = 41); acral lesions in 28%.
Fernández-Nieto 2020 [15]	Narrative review (n = 132)	-	To analyse and observe the predominance of dermatological lesions on the foot and hand.	Two different patterns of acral lesions; chilblains (72%) and erythema multiforme-like pattern. Acral lesions are predominantly on the toes and are rarely seen in other areas of the body.
Galván-Casas 2020 [15]	Prospective study (N = 375)	M = 153 W = 222	To describe the cutaneous manifestations of COVID-19 disease and relate them to other clinical findings.	Chilblains = 19%, urticarial lesions = 19% and maculopapular lesions = 47%, necrosis = 6%.
Duong 2020 [22]	Retrospective study (N = 219)	-	To establish the prevalence of dermatological lesions on the foot.	Chilblains are predominant as a dermatological sign in patients diagnosed with COVID-19.
Roca-Gines 2020 [23]	Case series study (N = 20)	1–18 M = 13 W = 7	To report skin lesions as possible symptoms of COVID-19 infection.	Dermatological lesions on hands and feet, being more predominant on the foot. Acral erythema = 30%, dactylitis = 20% maculopapular lesions = 35% and mixed pattern = 15%.
Marzano 2020 [24]	Multicentre case series study (n = 22)	60 M = 16 W = 6	To define the cutaneous manifestations of COVID-19.	Scattered papulovesicular lesions, with different symptoms and not predominantly on the foot but present on the trunk.
Zhang 2020 [25]	Case series study (N = 7)	59 M = 4 W = 3	To investigate the clinical and coagulation characteristics of patients with coronavirus critical illness 2019 (COVID-19) with acroischemia in the intensive care unit.	Acroischemia accompanied by cyanosis of the toes.
Piccolo 2020 [26]	Case series study (N = 63)	14 M = 27 W = 36	To report preliminary results on dermatological lesions in children.	Lesions on feet = 85.7%, and on feet and hands together = 7%. Foot lesions are predominant in children with suspected SARS-CoV-2 infection.
Massey 2020 [27]	Narrative review (n = 566)	-	To review the literature on chilblain-like skin lesions in the early stages of the global COVID-19 pandemic.	A multitude of chilblain-like skin manifestations on the foot by COVID-19.
Jamshidi 2021 [28]	Narrative review (n= 1847)	-	To assess the temporal relationship between different types of skin lesions and the severity of COVID-19.	Cutaneous manifestations = 5.95%. Patients with vascular lesions had the highest mortality rate.
Jiménez-Cebrian 2021 [9]	Review of reviews (n = 10)	-	To review the literature on the clinical manifestations of COVID-19 in the foot.	The most relevant manifestations were Kawasaki disease, acral perniosis lesions, pernio erythema and ischaemia, and necrosis.

## Data Availability

Not applicable.
